# Cardiovascular Response to Exercise with and Without Alcohol Consumption: Evidence of an Interaction Between Distance Covered and Perceived Exertion

**DOI:** 10.3390/nu18091407

**Published:** 2026-04-29

**Authors:** Thiago Ferreira de Sousa, Aline de Jesus Santos, José Carlos Aragão-Santos, Sandra Celina Fernandes Fonseca

**Affiliations:** 1 Department of Health Sciences, State University of Santa Cruz, Ilhéus 45662-900, BA, Brazil; prof.josecarlosaragao@gmail.com; 2 Department of Education, State University of Bahia, Teixeira de Freitas 45980-000, BA, Brazil; ajsantos.ppgef@uesc.br; 3 Research Center in Sports Sciences, Health Sciences and Human Development, Department of Sports, Exercise and Health Sciences, University of Trás-os-Montes and Alto Douro, 5000-801 Vila Real, Portugal; sfonseca@utad.pt

**Keywords:** physical activity, running, heart rate, alcohol consumption, RPE, young adults

## Abstract

Background: Acute alcohol consumption may interfere with the dynamics between internal and external load during exercise, potentially attenuating cardiovascular responses. Objective: This study investigated the association between distance covered during a running test and mean heart rate, while examining the moderating role of the rating of perceived exertion (RPE) under conditions with and without acute alcohol ingestion. Methods: This crossover experimental study included 12 physically active male university students (23.7 ± 3.7 years). Participants completed two intermittent running sessions (control and alcohol conditions), separated by ≥48 h. In the alcohol condition, participants consumed 0.4 g of ethanol/kg of body mass. Heart rate was continuously monitored using a Polar RCX5 monitor, and total distance covered and RPE (Borg 6–20 scale) were assessed immediately after test completion. Analyses included paired comparisons, Pearson correlations, and linear regression models with interaction terms. Results: No significant associations between variables were observed in the control condition. With alcohol consumption, distance covered was positively associated with mean heart rate, and RPE significantly moderated this relationship. Conclusions: Acute alcohol ingestion may modify the interaction between external load, perceived exertion, and cardiovascular response during running. These results highlight the importance of integrated monitoring of internal and external load, especially in contexts involving recent alcohol consumption.

## 1. Introduction

The cardiovascular response to running is a well-established indicator of internal load during physical exercise, as mean heart rate correlates with exercise intensity [1]. Rating of perceived exertion (RPE) has been validated across various exercise settings as a consistent predictor of exercise intensity and is related to physiological responses, including heart rate and blood lactate [2]. Furthermore, internal load estimated via RPE may differ among individuals with different levels of physical activity [3].

Combining external load measures, such as distance covered, with internal load measures, such as RPE and heart rate, has been advocated as a robust approach to characterizing exercise dosage in sport [4]. It is important to consider that internal load may vary by modality and individual physiological parameters [5], that RPE is associated with heart rate response during physical exercise [6], and that the transferability of RPE–physiological relationships from graded exercise tests to steady-state exercise remains uncertain [7]. However, gaps persist in understanding the relationship between external and internal load when contextual and behavioral factors influence these responses. In university populations, epidemiological studies indicate that physical activity and alcohol consumption often co-occur as lifestyle behaviors among young adults [8].

Beyond this behavioral co-occurrence, experimental evidence suggests that acute alcohol ingestion before exercise may influence physiological responses during physical exertion. Acute alcohol consumption has been associated with modifications in autonomic regulation and cardiovascular response, such as elevated nocturnal resting heart rate [9]. Alcohol ingestion reduces work performed above critical power during intense exercise, suggesting a direct impact on performance capacity [10]. In addition, moderate alcohol intake potentiates exercise-induced changes in metabolic markers such as blood lactate, indicating combined effects on effort physiology [11].

Yet little is known about how acute alcohol ingestion may influence the relationship between external load, perceived exertion, and cardiovascular responses during running, especially in outdoor exercise settings, which differ from the laboratory environment, in which climatic conditions are more controlled and variability due to the practice location is reduced, particularly the running surface. Moreover, the practical relevance of this topic may help inform guidelines for exercise load monitoring and practice by investigating an easily measurable external load indicator (i.e., distance covered during running) and a widely validated internal load measure (i.e., RPE) in relation to alcohol consumption and the cardiovascular response (i.e., heart rate). Thus, the present study aimed to estimate the effect of distance covered during running on mean heart rate, while accounting for the moderating role of perceived exertion under conditions with and without alcohol consumption, thereby contributing to a broader understanding of physiological responses to exercise under different behavioral conditions. We hypothesized that alcohol consumption would influence the relationship between heart rate and internal load during exercise.

## 2. Materials and Methods

### 2.1. Study Design

This study employed an experimental crossover design conducted under two experimental conditions: without alcohol consumption and with acute alcohol consumption. The protocol was approved by the Research Ethics Committee of the Federal University of Recôncavo da Bahia (UFRB) (approval number 2.771.693; approval date 16 July 2018) and complied with ethical principles for research involving human subjects. All participants were informed about the study’s objectives and procedures and signed an informed consent form prior to participation.

### 2.2. Participants

Twelve male university students aged 18 to 30 years participated in the study, selected through convenience sampling from students regularly enrolled at UFRB. Exclusion criteria consisted of: young adults engaging in less than 150 min per week of moderate-to-vigorous physical activity during leisure time, as self-reported via questionnaire [12]; those with problematic alcohol use, assessed using alcohol-dependence screening instruments [13,14]; and those with contraindications to physical activity, identified using the Physical Activity Readiness Questionnaire [15].

A post hoc statistical power analysis was conducted using G*Power software (version 3.1, Kiel, Germany). In the alcohol condition, the regression model showed a large effect size (R^2^ = 0.75; f^2^ = 3.0), yielding an estimated statistical power of approximately 0.96. In contrast, the model without alcohol consumption presented a small-to-moderate effect size (R^2^ = 0.098; f^2^ = 0.11), corresponding to a statistical power of approximately 0.13.

### 2.3. Procedures

Each participant completed two experimental sessions, under conditions with and without alcohol ingestion, separated by a minimum interval of 48 h. The order of the sessions was counterbalanced among participants. Prior to data collection, volunteers attended a familiarization session with the running test protocol and were instructed to avoid intense physical exercise, alcohol intake, and caffeine consumption during the 48 h preceding each session. Before the familiarization session, the volunteers were assessed for anthropometric variables (body mass and height). Body mass was measured using a digital scale (Britânia, Joinville, Brazil) with a capacity of 150 kg, following standard anthropometric procedures. Height was measured using a wall-mounted stadiometer with a precision of 0.1 cm. Body mass index (BMI) was calculated as body mass in kilograms divided by height in meters squared (kg/m^2^), according to standard anthropometric guidelines. Data collection was conducted in September 2018, consistently at 10:00 a.m. Physical assessments were conducted in a covered sports court. Ambient temperature in degrees Celsius (°C) was monitored using a digital thermometer (model AK28, Akso, São Leopoldo, Brazil), with an average temperature of 24 °C.

In the experimental condition with alcohol consumption, participants ingested an acute, moderate dose of alcohol (vodka, Smirnoff brand, Itaitinga, Brazil), corresponding to 0.4 g of ethanol per kilogram of body mass, which represents half of a dose considered high [16], diluted in artificial orange juice (ratio 1:3.2; beverage alcohol content equivalent to 23.8%). Alcohol administration was standardized. In the control condition, participants ingested only the juice, with no added alcohol.

After ingesting the beverage corresponding to the experimental condition, participants remained at rest for approximately 30 min, the estimated time required for alcohol absorption prior to the physical test [17]. Blood samples were collected to determine pre- and post-test lactate and glucose concentrations. The test was initiated only if resting blood lactate concentration was <4 mmol/L [18] and blood glucose concentration was >70 mg/dL, measured using a portable lactate analyzer (Accutrend Plus, Roche, Basel, Switzerland), with lactate reported in mmol/L and glucose in mg/dL. After completing the test, participants proceeded to a nearby blood collection area, where blood was drawn again to measure lactate and glucose levels.

### 2.4. Instruments

Physical performance was assessed using the Yo-Yo Intermittent Recovery Test, performed over a 20 m course with progressively increasing speed controlled by standardized auditory signals. The test was terminated when the participant failed to reach the required distance within the allotted time on two consecutive attempts [19]. Throughout the test, heart rate was continuously monitored via a heart rate monitor (Polar RCX5, Polar, Kempele, Finland). The mean heart rate obtained during the test was taken as the dependent variable, representing the cardiovascular response to physical exertion. The independent variables analyzed were total distance covered during the running test, expressed in meters and used as an indicator of external load, and RPE, assessed at rest 30 min after test completion using the Borg scale [20], as recommended by Foster et al. [21], and used as an internal load indicator.

### 2.5. Data Analysis

Data were tabulated in a spreadsheet and analyzed using SPSS software (version 25.0, Chicago, IL, USA). Initially, data normality was assessed using the Shapiro–Wilk test, complemented by Skewness and Kurtosis (with values between −2 and 2 considered satisfactory). Analyses were performed separately for each experimental condition (with and without alcohol consumption). Paired *t*-tests were performed to compare the primary variables (distance covered, mean heart rate, and RPE) and the secondary variables (blood lactate and glucose concentrations) across the alcohol and control conditions. Correlations between the independent variables and mean heart rate were estimated using Pearson’s correlation coefficient. Additionally, Spearman correlation analyses were conducted to assess the robustness of the results, given the small sample size and potential deviations from normality. Subsequently, Multiple Linear Regression was applied, including an interaction term between distance covered and RPE, to estimate their effects on mean heart rate. Distance and RPE were mean-centered prior to creating the interaction term in order to reduce multicollinearity and enhance the interpretability of regression coefficients. Multicollinearity was assessed using the variance inflation factor (VIF). Residual diagnostics were examined to evaluate the assumptions of the regression models, and normality of residuals was assessed using histograms and normal P–P plots, with no substantial deviations from normality observed. The significance level was set at 5%.

## 3. Results

### 3.1. Sample Characteristics, Data Distribution, and Paired Comparisons

Twelve university students participated in this study. The mean age was 23.67 years (SD: 3.68; minimum: 19; maximum: 30). Participants had a mean body mass of 73.65 kg (SD: 8.14) and a mean BMI of 24.33 kg/m^2^ (SD: 3.65). Table 1 presents the descriptive statistics and comparison of metabolic variables across alcohol consumption and control conditions. No statistically significant differences in the mean levels of metabolic variables were observed between alcohol consumption and control conditions. Blood lactate increased markedly from pre- to post-test in both experimental conditions, whereas blood glucose levels remained relatively stable.

Most variables (distance covered, RPE, and mean heart rate) showed acceptable distribution values within the range of −2 to 2 for skewness and kurtosis (*p*-value > 0.05 of the Shapiro–Wilk test). The only variable that slightly deviated from normality was distance covered in the condition without alcohol consumption (*p* = 0.047). Considering the small sample size and the approximate symmetry of the distribution, parametric analyses were retained. Table 2 displays the descriptive statistics and paired comparisons for the variables under the conditions without and with alcohol consumption. Paired comparisons between experimental conditions showed no significant differences in distance covered (*p* = 0.765), RPE (*p* = 0.476), or mean heart rate (*p* = 0.358).

Table 3 presents the correlations of distance covered during the test and RPE with mean heart rate under conditions with and without alcohol consumption. A positive correlation was observed between distance covered and mean heart rate in the condition with alcohol consumption. To assess the robustness of the findings, given the small sample size and the slight deviation from normality observed for distance in the condition without alcohol consumption, additional analyses using Spearman correlations (rho) were conducted. These analyses produced results consistent with those obtained using Pearson correlations. Specifically, in the alcohol condition, distance covered remained significantly associated with mean heart rate (rho = 0.616, *p* = 0.033), whereas no significant correlations were observed in the condition without alcohol consumption.

### 3.2. Effects on Mean Heart Rate

Table 4 provides the regression coefficients for the effects of distance covered and RPE, along with their interaction term, on mean heart rate under each experimental condition. In the condition without alcohol consumption, no effects were observed among the variables. For each additional meter above the mean distance, mean heart rate increases by 0.032 beats per minute when RPE is at its mean value. RPE alone was not associated with mean heart rate when distance was at its mean value. The relationship between distance and mean heart rate changes depending on RPE (*p*: 0.036).

### 3.3. Interaction Between Distance and RPE on Heart Rate Under Alcohol Consumption

**Figure 1:**
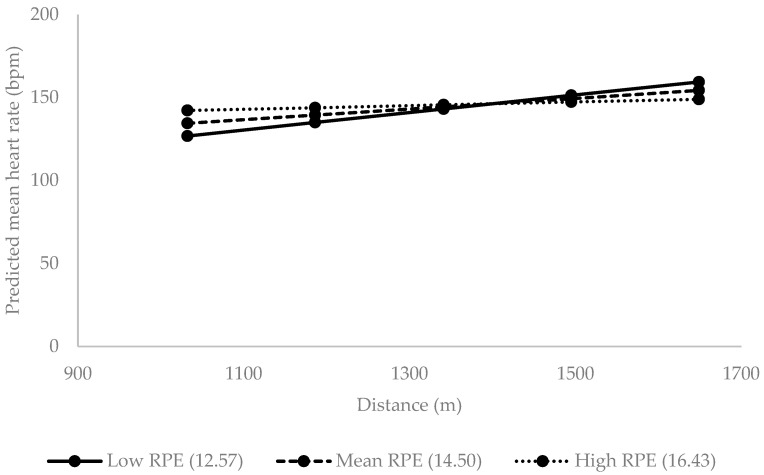
Predicted mean heart rate (bpm: beats per minute) across running distance under alcohol consumption for representative levels of perceived exertion (RPE): low (mean − 1 SD = 12.57), mean (14.50), and high (mean + 1 SD = 16.43). Brazil. 2018.

It was observed that RPE showed an interaction in the effect of distance covered on heart rate (Table 4). Predicted regression slopes were plotted for representative levels of perceived exertion (mean − 1 SD, mean, and mean + 1 SD) to illustrate the interaction between distance and RPE on mean heart rate under alcohol consumption (Figure 1).

## 4. Discussion

Our main finding was the association between distance covered and mean heart rate moderated by RPE under the influence of alcohol. Specifically, in the control condition (without alcohol consumption), there was no significant effect of distance covered and RPE with respect to mean heart rate. This result suggests that, under normal physiological conditions, the external load represented by distance covered may not be sufficient to explain the variability in cardiovascular responses during progressively intense exercise in young adults, although these external and internal measures demonstrate positive effects [4,22]. The relationship between external and internal load may be attenuated when individuals adjust their exercise pace [23]; however, this pattern was not applied in this experimental design.

The absence of a significant effect of RPE on mean heart rate in the condition without alcohol reinforces previous findings indicating that RPE possibly does not exclusively reflect central physiological responses but rather results from a complex integration of cardiorespiratory, metabolic, and psychosocial signals [3,7,20,24]. The substantial increase in blood lactate observed after the test in the present study across both experimental conditions also supports the high metabolic demand imposed by the Yo-Yo intermittent running protocol. Furthermore, our experiment focused on intermittent running, which favors a significant increase in intensity and thus may not have generated linear effects on heart rate. Recent studies demonstrate that, during continuous exercise, the relationship between RPE and heart rate may be moderated in different groups with or without disease [1,24], being directly influenced by exercise intensity and duration, sport modality, training level, psychological factors, and environmental and contextual variables (e.g., mental fatigue, heat, music, and motivation) [24].

Paired comparisons between experimental conditions did not reveal significant differences in distance covered, RPE, or mean heart rate. This finding suggests that the effects observed under alcohol consumption were not driven by overall differences in performance or physiological responses between conditions, but rather by changes in the relationship between external load and cardiovascular response. In contrast, under the condition of alcohol consumption, a significant positive effect of distance covered on mean heart rate was observed, indicating a potentially greater cardiovascular demand as external load increased. These findings align with studies showing that acute alcohol consumption contributes to lower test performance and reduced peak power output [25], as well as increased heart rate during exercise, for instance, cycling at 50% of maximal oxygen uptake [26]. Conversely, no differences in RPE have been observed between conditions with and without alcohol consumption [26]. Additionally, alcohol may impair cardiovascular control mechanisms, facilitating attenuations in heart rate and effects associated with atrial fibrillation patterns [27].

Regression analysis results further indicated that, under the influence of alcohol, distance covered showed positive effects on mean heart rate, whereas RPE was not significant. On the other hand, alcohol may intensify metabolic responses to exercise, such as increased blood lactate concentration, which could, theoretically, increase subjective effort perception; however, the effect of alcohol on lactate is not conclusive, even at different intensities [11], as also observed in our previous analysis [28]. Regardless, the greater relevance of RPE observed in the present study may directly reflect a state of heightened physiological stress under the condition of alcohol consumption [28] when moderating the effect of external load on the cardiovascular response.

The presence of a significant interaction effect between distance and RPE in the alcohol condition suggests that perceived exertion could modulate the relationship between external load and cardiovascular response, indicating a possible impairment of exercise self-regulation capacity at higher intensities. This finding is consistent with the conceptualization of RPE as a central integrative signal in effort control, particularly in contexts where behavioral and contextual factors modulate physiological responses to exercise [20,29]. RPE as an indicator of internal load may be affected by physiological changes induced by substances such as alcohol [26]. From a practical standpoint, these results suggest that, under conditions of alcohol consumption, the association between external workload and heart rate may become attenuated as perceived exertion increases.

Thus, the integrated use of internal load indicators, such as RPE, in effort control strategies in recreational contexts, where individuals may engage in exercise associated with alcohol consumption, appears relevant. In young populations, such as university students, an association has been observed between alcohol consumption and physical activity practice, which varies according to the level of analysis, given that it is possible that college students with higher average levels of exercise also exhibit higher average alcohol consumption, and on days when they exercise more than usual, their alcohol intake may be lower [30,31]. Therefore, health professionals and those working in sports and recreational centers, including hotels and clubs, should consider recent alcohol consumption history when prescribing and/or advising physical activity practices, especially those of higher intensity or longer duration.

Among the limitations of the present study, the small sample size should be highlighted. This aspect may be partly related to the requirement of alcohol ingestion in one of the experimental conditions, which may have discouraged adherence to the protocol. Nevertheless, strategies for broad dissemination were adopted, along with a detailed explanation of the intervention procedures, including their objectives, potential risks, and benefits. In addition, the small sample size limited the possibility of applying more complex statistical approaches, such as generalized linear mixed models, which could account for the within-subject correlation between experimental conditions and allow the inclusion of additional predictors in the regression analyses. Due to the exploratory nature of this study and the limited sample size, analyses were conducted separately for each condition to examine the relationships among variables within each experimental context.

Furthermore, the amount of alcohol administered constitutes another limitation, as it may not accurately represent cultural consumption patterns associated with physical activity among young men, nor did it estimate the degree of direct influence in terms of intoxication. Additionally, the study did not include objective verification of alcohol exposure, such as breath or blood alcohol concentration measurements, which may introduce inter-individual variability in the physiological responses observed after alcohol ingestion. Nevertheless, the dose used was classified as moderate and defined based on safety criteria and ethical principles, in order to preserve participants’ health, since excessive ethanol intake is associated with impaired physical performance [32].

It is worth noting that the use of the Yo-Yo intermittent test as the external-load context is less common than progressive continuous aerobic tests, but in our view, it is a typical test for young adults. The use of mean heart rate as the sole indicator of internal load represents another limitation, as this measure may be influenced by factors such as test duration and distance covered during aerobic tests. Finally, the lack of control over pre-existing mental fatigue and individual alcohol tolerance is a recognized limitation, since these factors could have repercussions on potentially negative results in running performance and the degree of intoxication, respectively.

Future research should include larger and more diverse samples to enhance the generalizability of findings and incorporate objective measurements of blood alcohol concentration to verify actual exposure levels. Further studies should also investigate the effects of different alcohol doses and varying intervals between alcohol ingestion and exercise, as these factors may distinctly influence cardiovascular and perceptual responses. In addition, examining individuals with diverse characteristics, such as sex, age, fitness level, alcohol tolerance, and habitual drinking patterns, while including additional physiological indicators may contribute to a more comprehensive understanding of how alcohol influences physiological and perceptual responses during exercise.

## 5. Conclusions

In our sample of university students, using a crossover design, the data suggest that acute alcohol ingestion may modify the association between external load and mean heart rate, with perceived exertion influencing this relationship. Thus, these findings indicate that mean heart rate responses may exhibit variability following alcohol consumption. We emphasize that confirmation in larger, adequately powered studies with objective measures of alcohol intoxication is warranted to contribute to a more comprehensive understanding of physiological responses to physical exercise in contexts of alcohol consumption, with relevant implications for the safe practice of physical activity in young adults.

## Figures and Tables

**Table 1:** Descriptive statistics and paired comparisons of metabolic variables under conditions with and without alcohol consumption in young adults. Brazil. 2018.

Variables	Without Alcohol Consumption				with Alcohol Consumption				
	S	K	*p*	Mean (SD)	S	K	*p*	Mean (SD)	Paired *t*-Test
Blood lactate (mmol/L), pre-test	−0.058	−1.151	0.778	2.65 (0.59)	1.136	0.614	0.112	2.45 (0.79)	0.665
Blood lactate (mmol/L), post-test	1.445	3.697	0.071	11.08 (3.90)	1.081	0.804	0.141	11.01 (2.76)	0.455
Blood glucose (mg/dL), pre-test	−0.002	−0.613	0.720	93.25 (9.75)	0.615	−1.199	0.079	94.58 (5.74)	0.493
Blood glucose (mg/dL), post-test	1.307	0.961	0.035	92.83 (11.31)	−0.111	−1.148	0.786	89.67 (8.80)	0.962

S: Skewness; K: Kurtosis; *p*: *p*-value of the Shapiro–Wilk test; mmol/L: millimoles per liter; mg/dL: milligrams per deciliter; SD: Standard Deviation.

**Table 2:** Descriptive statistics and paired comparisons of exercise variables under conditions with and without alcohol consumption in young adults. Brazil. 2018.

Variables	Without Alcohol Consumption Mean (SD)	with Alcohol Consumption Mean (SD)	Paired *t*-Test
Distance (m)	1388.33 (336.12)	1340.83 (308.85)	0.765
RPE	13.75 (2.73)	14.50 (1.93)	0.476
Mean heart rate (bpm)	147.58 (9.50)	143.17 (10.41)	0.358

m: meters; SD: Standard Deviation; RPE: Rating of Perceived Exertion; bpm: beats per minute.

**Table 3:** Correlations of distance covered during running and perceived exertion with mean heart rate (beats per minute) in young adults. Analyses considering conditions with and without alcohol consumption. Brazil. 2018.

Variables	r	*p*
Without alcohol consumption		
Distance (m)	0.099	0.761
RPE	−0.239	0.455
with alcohol consumption		
Distance (m)	0.735	0.006
RPE	0.045	0.889

r: Pearson correlation; m: meters; RPE: Rating of Perceived Exertion.

**Table 4:** Regression coefficients (β) of distance covered during running and perceived exertion, both as mean-centered, on mean heart rate in young adults. Analyses considering conditions with and without alcohol consumption. Brazil. 2018.

Variables	β (95% CI)	*p*	VIF	R^2^	R^2^ Adj.	F-Test	*p*-Value
Without alcohol consumption				0.098	−0.241	F(3,8) = 0.288	0.833
Distance (m)	0.002 (−0.021, 0.024)	0.847	1.054				
RPE	−0.772 (−3.485, 1.942)	0.530	1.017				
Distance × RPE	0.002 (−0.008, 0.012)	0.626	1.066				
with alcohol consumption				0.750	0.656	F(3,8) = 8.00	0.009
Distance (m)	0.032 (0.017, 0.048)	0.001	1.253				
RPE	0.647 (−1.877, 3.171)	0.571	1.320				
Distance × RPE	−0.011 (−0.021, −0.001)	0.036	1.581				

β: slope coefficient; 95% CI: 95% confidence interval; m: meters; RPE: Rating of Perceived Exertion; Adj.: Adjusted; VIF: Variance Inflation Factor; R^2^: Coefficient of determination.

## Data Availability

Dataset available on request from the authors. The data are not publicly available due to privacy and ethical restrictions.

## Abbreviations

The following abbreviations are used in this manuscript:

RPE	Rating of Perceived Exertion
UFRB	Federal University of Recôncavo da Bahia
